# Centre of pressure during walking after unilateral transfemoral amputation

**DOI:** 10.1038/s41598-022-22254-5

**Published:** 2022-10-19

**Authors:** Daisuke Ichimura, Genki Hisano, Hiroto Murata, Toshiki Kobayashi, Hiroaki Hobara

**Affiliations:** 1 Artificial Intelligence Research Center, National Institute of Advanced Industrial Science and Technology (AIST), Tokyo, Japan; 2 Department of Systems and Control Engineering, Tokyo Institute of Technology, Tokyo, Japan; 3 Japan Society for the Promotion of Science (JSPS), Tokyo, Japan; 4 Department of Mechanical Engineering, Tokyo University of Science, Chiba, Japan; 5 Department of Biomedical Engineering, Faculty of Engineering, The Hong Kong Polytechnic University, Hong Kong, China; 6 Faculty of Advanced Engineering, Tokyo University of Science, Tokyo, Japan

**Keywords:** Biomedical engineering, Rehabilitation

## Abstract

Lower-limb amputation imposes a health burden on amputees; thus, gait assessments are required prophylactically and clinically, particularly for individuals with unilateral transfemoral amputation (UTFA). The centre of pressure (COP) during walking is one of the most useful parameters for evaluating gait. Although superimposed COP trajectories reflect the gait characteristics of individuals with neurological disorders, the quantitative characteristics based on the COP trajectories of individuals with UTFA remain unclear. Thus, these COP trajectories were investigated across a range of walking speeds in this study. The COP trajectories were recorded on a split-belt force-instrumented treadmill at eight walking speeds. Asymmetry and variability parameters were compared based on the COP trajectories of 25 individuals with UTFA and 25 able-bodied controls. The COP trajectories of the individuals with UTFA were significantly larger in lateral asymmetry and variability but did not show significant differences in anterior–posterior variability compared with those of the able-bodied controls. Further, the individuals with UTFA demonstrated larger lateral asymmetry at lower speeds. These results suggest that (1) individuals with UTFA adopt orientation-specific balance control strategies during gait and (2) individuals with UTFA could also be exposed to a higher risk of falling at lower walk speeds.

## Introduction

Lower-limb amputation imposes a burden on the daily lives of amputees, resulting in problems such as gait deterioration and subsequent limited mobility and poor quality of life^1,2^. In particular, individuals with unilateral transfemoral amputation (UTFA) are exposed to a high risk of falls^3,4^ and asymmetric gait, which increases the risk of secondary joint disorders^5,6^. Thus, gait assessments for individuals with UTFA are indispensable not only for providing quantitative information to help prevent injuries and prescription of treatments, but also for monitoring rehabilitation progress with prosthetic variation.

Most gait assessments require considerable time to attach markers and set up devices, as well as to analyse extensive numerical data laid out in tables. However, regular gait assessments in clinical practice and daily life require ease of measurement and interpretation. Centre of pressure (COP) trajectories during walking represent summarised gait features individually, enabling markerless, unconstrained, and time-efficient gait assessments^7^. The COP is the point location of the vertical ground reaction force vector that represents a weighted average of all the pressures over the surface of the area in contact with the ground^8^. Thus, the moments generated by vertical ground reaction force are null at this point on the ground plane. The COP is knowns as one of the biomechanical variables which evaluate static or dynamic balance control. For example, previous studies have reported that individuals with UTFA have asymmetric COP trajectories between the intact and prosthetic legs during a single-limb stance phase using separate force plates on each side^9,10^. Furthermore, it has been reported that COP trajectories are related to the severity of neurological disorders^11–13^. Although an instrumented treadmill enables COP trajectory detection over multiple gait cycles in individuals with UTFA^7^, the quantitative characteristics corresponding to COP trajectories remain unclear.

The objective of the present study was to investigate COP trajectories across a range of walking speeds in individuals with UTFA. According to a previous study, the intact leg contributes to lateral stability during gait, which indicates different gait principles compared to those of able-bodied controls^9,10^. Therefore, we hypothesised that individuals with UTFA would show larger asymmetry and variability in the lateral components of COP trajectories than able-bodied controls during walking.

## Methods

### Participants

Twenty-five individuals with UTFA were recruited (Table 1, Table S1 in the Supplementary Information). The aetiology of amputation included trauma, sarcoma, cancer, and congenital. All individuals with UTFA used their habitual mechanical or microprocessor-controlled prosthetic knees and mechanical feet. The inclusion criteria for individuals with UTFA were (1) no neuromuscular disorder or complications, (2) no lower-limb functional limitations that severely interfere with their daily activities, and (3) functional classification level of K3 or K4 and ability to walk without using external aids or supports. Furthermore, we selected 25 sex-, age-, body-height-, body-mass-, and preferred-walking-speed-matched able-bodied controls who were not significantly different from those of individuals with UTFA (Table 1). Before the experiment, all participants provided written informed consent as approved by the local ethics committee. The study was approved by the review board of our institution (Environment and Safety Headquarters, Safety Management Division, National Institute of Advanced Industrial Science and Technology) and conducted according to the Declaration of Helsinki guidelines.

**Table 1:** Characteristics of able-bodied controls and individuals with UTFA (mean ± standard deviation). The preferred walking speed was determined by gradually increasing the speed of the treadmill (from 2.0 km/h) until the subjects indicated that the walking velocity felt comfortable.

	Able-bodied controls	Individuals with UTFA
Number (female)	25 (5)	25 (6)
Age (years)	28.52 ± 8.93	31.52 ± 10.07
Body height (m)	1.68 ± 0.63	1.66 ± 0.74
Body mass (kg)	67.94 ± 11.38	65.77 ± 13.96
Preferred walking speed (km/h)	4.13 ± 0.45	4.04 ± 0.75
Time since amputation (years)	–	12.18 ± 9.10
Prosthesis in NMPK	–	14
Prosthesis in MPK	–	11

Abbreviation: *NMPK*, non-microprocessor knee; *MPK*, microprocessor knee.

### Experimental procedure and data collection

**Figure 1:**
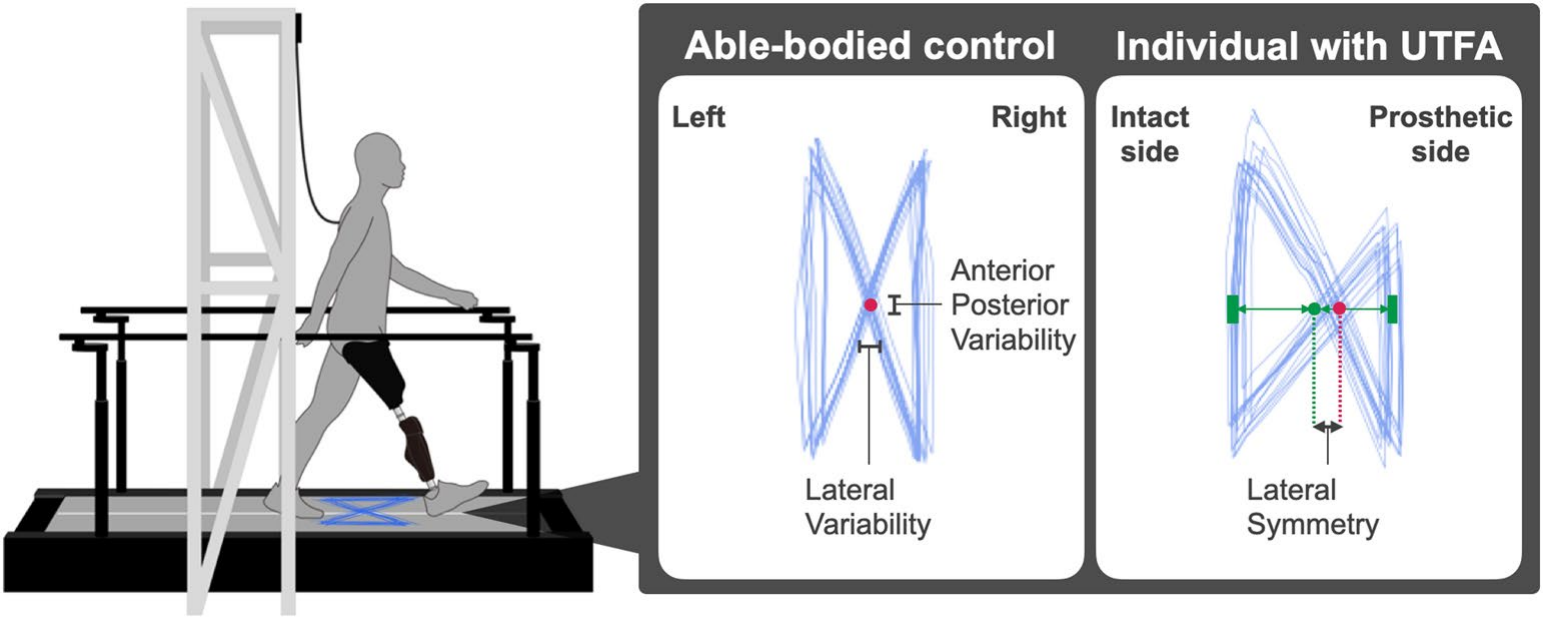
Schematic representation of the COP trajectory called a “butterfly diagram” derived from a split-belt force-instrumented treadmill. The treadmill was equipped with a safety harness and two handrails to prevent falling. The right panels show the COP trajectories recorded from an able-bodied control and individual with UTFA (right-limb amputation). The straight lines represent the single support stance phase, and the diagonal line represents the double support stance phase^7^. The red circles indicate the average intersection positions of the COP trajectories during walking. The average position of the straight lines in the lateral direction is the green square. The midpoint between the left and right green squares is the green circle which indicates the perfect lateral symmetry positions of the COP trajectories during walking. Lateral symmetry is defined as the lateral distance between the red and green circles^11,13^.

Previous studies have shown that differences in gait parameters between treadmill and overground walking were negligible^14,15^; accordingly, we used an instrumented treadmill for gait analysis in line with similar studies^5,11,13^. All participants walked for 30 s at eight different speeds (2.0, 2.5, 3.0, 3.5, 4.0, 4.5, 5.0, and 5.5 km/h) on a split-belt force-instrumented treadmill (Fig. 1; FTMH-1244WA, Tec Gihan, Kyoto, Japan). During all trials, a safety harness was used to prevent falls and relieve the fear of falling in the participants. We ensured that the harness was applied with adequate slack to prevent it from influencing natural walking. Based on a previous study^5^, all participants performed an adaptation trial for at least 7 min to become accustomed to treadmill walking before data collection. In the adaptation trial, participants became familiar with all experimental speeds, and we confirmed that they could walk at each speed for 30 s with confidence. Finally, we set an adequate rest time between trials for all participants.

### Data analysis

Ground reaction force (GRF) data from the instrumented treadmill were converted into medio-lateral and anterior–posterior COP data sampled at 1000 Hz, and a 20-Hz low-pass, fourth-order, zero-lag Butterworth filter was applied. We determined the timing of foot contact and toe-off for both limbs by using a vertical GRF threshold of 40 N^5^, based on which strides were calculated. Then, we used 15 strides after 2 s of achieving each walking speed to analyse the COP data.

The COP trajectory during walking was produced in a graphical pattern as a “butterfly diagram” (Fig. 1). Afterwards, we determined three key gait parameters from the COP butterfly diagram^11,13^: lateral symmetry (LS; the left/right shift of the intersection point, where “zero position” is equivalent to perfect symmetry), lateral variability (LV; the standard deviation of the intersection point in the lateral direction, where “zero” is equivalent to constant strides in terms of width between the leg), and anterior–posterior variability (APV; the standard deviation of the intersection point in the anterior–posterior direction, where “zero” is equivalent to constant strides while walking on the treadmill). These parameters, which can be used to assess continuous COP trajectories with multiple strides, reflect the overall movements of individuals throughout the gait cycle.

### Statistics

As our data were not normally distributed (Shapiro–Wilk test, *p* < 0.05), we used non-parametric tests for all statistical analyses. Mann–Whitney U tests were conducted for paired comparisons between individuals with UTFA and able-bodied controls. We also performed the Friedman test to investigate the main effect of walking speed in each group. When a significant main effect was observed, the Wilcoxon signed-rank test was conducted as a post-hoc comparison. Statistical significance was set at *p* < 0.05 and adjusted with the Bonferroni correction. All statistical comparisons were performed using RStudio version 1.4.1717 (RStudio, Inc.). The users of microprocessor and non-microprocessor knees in the above analyses were compared, and the results are presented in the Supplementary Information (Figure S1, Table S2).

## Results

**Figure 2:**
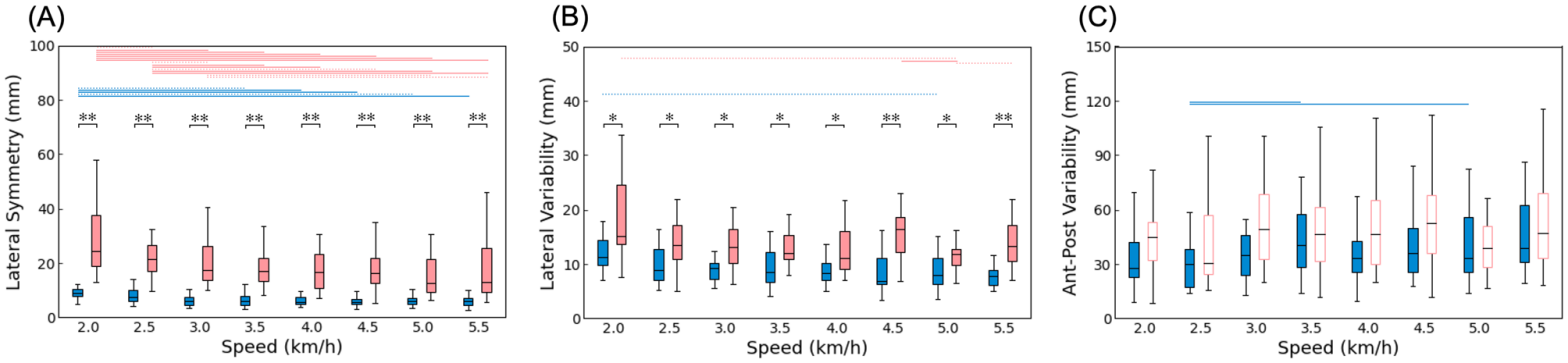
Whisker-box plots of lateral symmetry (**A**), lateral variability (**B**), and anterior–posterior variability (**C**) at eight different walking speeds. The blue and red boxes represent able-bodied controls and individuals with UTFA, respectively. The asterisks indicate significant differences between the able-bodied controls and individuals with UTFA (*p < 0.05, **p < 0.01). Significant and non-significant main effects of walking speeds are indicated by filled and unfilled boxes, respectively. The blue (able-bodied controls) and red (individuals with UTFA) horizontal lines indicate significant differences between walking speeds (dotted line: *p* < 0.05, solid line: *p* < 0.01).

In Fig. 2A, the individuals with UTFA exhibit significantly larger LS than the able-bodied controls across all speeds (*p* < 0.05). Furthermore, there are significant main effects of walking speed on LS in both individuals with UTFA and the able-bodied controls (*p* < 0.05). Post-hoc tests revealed that the LS values corresponding to low walking speeds (especially 2.0, 2.5, and 3.0 km/h in individuals with UTFA) were significantly greater than those obtained at other walking speeds (*p* < 0.05).

Figure 2B demonstrates that the LV of the individuals with UTFA is significantly larger than that of the able-bodied controls across all speeds (*p* < 0.05). In contrast, no significant difference in APV is observed between the two groups at any speed (Fig. 2C). The LV at 2.0 km/h is greater than that at higher walking speeds (Fig. 2B). A significant main effect of walking speed on APV is also observed in the able-bodied controls (*p* < 0.05) but not in the individuals with UTFA (Fig. 2C).

## Discussion

The objective of the present study was to investigate COP trajectories across a range of walking speeds in individuals with UTFA. As shown in Fig. 2A and B, the LS and LV in the COP butterfly diagram for the individuals with UTFA are significantly greater than those of the able-bodied controls. However, there is no significant difference in APV between the two groups over a wide range of walking speeds (Fig. 2C). These results support our hypothesis that individuals with UTFA show larger asymmetry and variability in the lateral components of COP trajectories than able-bodied controls during walking.

One possible explanation for the large LS and LV in subjects with UTFA may be a compensatory strategy for lateral instability during walking. According to a previous study, the prosthetic leg cannot control the lateral moment of the ankle, which causes instability of the stance phase^9^. Consequently, the intact legs of individuals with UTFA must compensate for the lateral instability derived from their prosthetic legs, leading to lateral asymmetry of the COP trajectories^9,10^. In addition, Lin et al.^16^ reported that step width variability, which is related to the LV, was positively correlated with the functional capacity of physical activity in lower-limb amputees. Thus, the greater LV in our study may reflect the ability of individuals with UTFA with higher physical activity levels (K3 and K4). As there were no significant differences in APV between the individuals with UTFA and the able-bodied controls (Fig. 2C), the current results suggest that individuals with UTFA adopt orientation-specific balance control strategies during gait.

It is worth noting that the individuals with UTFA exhibit larger LS at lower speeds (2.0 and 2.5 km/h) compared to the other conditions (Fig. 2A). These results suggest that they modulate their dynamic body balance, specifically at lower speeds. As a slower gait and its related gait variation are associated with falls in individuals with neurological disorders^17^, the current results indicate that individuals with UTFA may also be exposed to a higher risk of falling while walking at lower speeds. These results suggest that a specific range of walking speeds may be associated with a low risk of falling for individuals with UTFA.

As shown in Fig. 2C, no significant differences in APV was observed between the individuals with UTFA and the able-bodied controls. A previous study reported that APV was associated with ataxia severity in individuals with multiple sclerosis^11^. Ataxia in multiple sclerosis progresses to bilateral disorders^18^, while individuals with UTFA have unilateral disabilities. In contrast, the LS and LV of individuals with UTFA were significantly greater than those of able-bodied controls over a wide range of walking speeds (Fig. 2A,B). A greater lateral COP component deviation during walking has also been observed in individuals with Parkinson's disease^13^. Interestingly, Parkinson’s disease often induces unilateral motor impairment, leading to asymmetric gait parameters, such as the swing time between the affected and unaffected limbs^19^. Therefore, as COP butterfly diagrams are indicative of uni- or bilateral functional gait impairments, individuals with UTFA and Parkinson’s disease may benefit from a similar gait rehabilitation program.

Measuring the COP butterfly diagram is useful for gait assessment in individuals with UTFA. The COP trajectories represent the overall gait visually, facilitating measurement and interpretation in routine clinical practice^13^. In particular, the parameters used in this study enable quantitative gait assessment, which has several benefits for individuals with UTFA. For example, if various daily walking data in addition to COP could be obtained, we could propose a safe environment, where the risk of falling is low, based on the analysis of COP data. This proposal has the potential to reduce the risk of falls by continuously measuring COP, which could be relatively easy to perform in a clinical setting and provide the results to individuals with UTFA. Further, quantitative assessment of COP parameters during gait could facilitate the selection of appropriate prosthetic components, clarify the rehabilitation progress, and facilitate the setting of realistic gait training objectives^7^, contributing to the motivation of individuals with UTFA and healthcare professionals using objective feedback. Therefore, COP butterfly diagrams can be valuable gait assessment tools in individuals with UTFA. In particular, the LS and LV in our study could be adopted as standards for interventions such as gait training or component replacement if they deviate from these values, in addition to simple gait asymmetry values, in individuals with UTFA who are active (K3 or K4). The mean values and standard deviations of these parameters are described in the Supplementary Information (Table S3).

There are certain concerns and limitations regarding the interpretation of our study. First, we recruited relatively young (30.3 ± 9.0 years) individuals with UTFA at functional levels K3 or K4. However, previous studies have reported that gait patterns vary with age^20^ and K-level^21^. Thus, individuals with a wide range of ages and functional levels must be recruited in future studies. Second, we did not control the prosthetic components, such as the prosthetic knee and foot, as described in the Supplementary Information (Table S1). As prosthetic components affect the gait symmetry of individuals with UTFA^22^, the COP trajectories and gait characteristics of individuals with UTFA should be comprehensively investigated in future studies. Third, we did not collect motion data with the COP simultaneously to compute a local frame associated with each individual. Therefore, motion data must be obtained to identify a coordinate frame for the COP that allows for a better gait characterization in future work.

## Conclusion

Individuals with UTFA exhibited significantly larger LS and LV compared to able-bodied controls. However, there was no significant difference in APV between the two groups. Further, individuals with UTFA demonstrated larger LS at lower speeds (2.0 and 2.5 km/h). These results suggest that (1) individuals with UTFA adopt orientation-specific balance control strategies during gait and (2) individuals with UTFA may also be exposed to a higher risk of falling while walking at relatively low speeds. Thus, the quantitative COP analysis presented in this study would be useful for clinical gait assessment.

## Supplementary Information

Supplementary Information 1.

Supplementary Information 2.

Supplementary Information 3.

## Data Availability

The datasets used and/or analysed during the current study available from the corresponding author on reasonable request.
