# Supplementary material for: Centre of pressure during walking after unilateral transfemoral amputation
Source: Sci Rep. 2022 Oct 19;12:17501. doi: 10.1038/s41598-022-22254-5 (PMC9582189; doi:10.1038/s41598-022-22254-5)
Supplement: Supplementary file 2 — Supplementary Information 2. [file 41598_2022_22254_MOESM2_ESM.docx]

**Comparisons of asymmetry and variability parameters based on centre of pressure trajectories between a microprocessor knee and a non-microprocessor knee**

Eleven individuals with unilateral transfemoral amputation (UTFA) using microprocessor knees (MPKs) and 14 individuals with UTFA using a non-microprocessor knee (NMPK) were involved in the analysis. The two groups were matched in terms of sex, age, body height, body mass, preferred walking speed, and time since amputation (Table S2). We compared the asymmetry and variability parameters based on the centre of pressure (COP) trajectories between the two groups (detailed in Section 2).

The statistical analysis results reveal that there are no significant differences in lateral symmetry (LS), lateral variability (LV), or anterior-posterior variability (APV) between the two groups over a wide range of walking speeds (Figure S1). There is a significant main effect of walking speed for MPK users on LS (p < 0.05) (Figure S1-A), but not on LV (Figure S1-B) or APV (Figure S1-C). In addition, there is a significant main effect of walking speed on LS, LV, and APV in NMPK users (p < 0.05). The results of post-hoc tests reveal significant differences in LV at 4.5 vs 5.0 km/h (Figure S1-B), APV at 2.5 vs 5.5 km/h (Figure S1-C), and APV at 5.0 vs 5.5 km/h (Figure S1-C) in NMPK users.

These results suggest that the availability of MPK/NMPK has little effect on the COP trajectories and the related gait parameters during walking in individuals with UTFA. However, there are certain concerns and limitations regarding the interpretation of this analysis, such as the limited recruitment of high activity subjects (K3, K4) and the lack of uniformity in the prosthetic components.

**Table S2**. Characteristics of MPK and NMPK users (mean ± standard deviation).


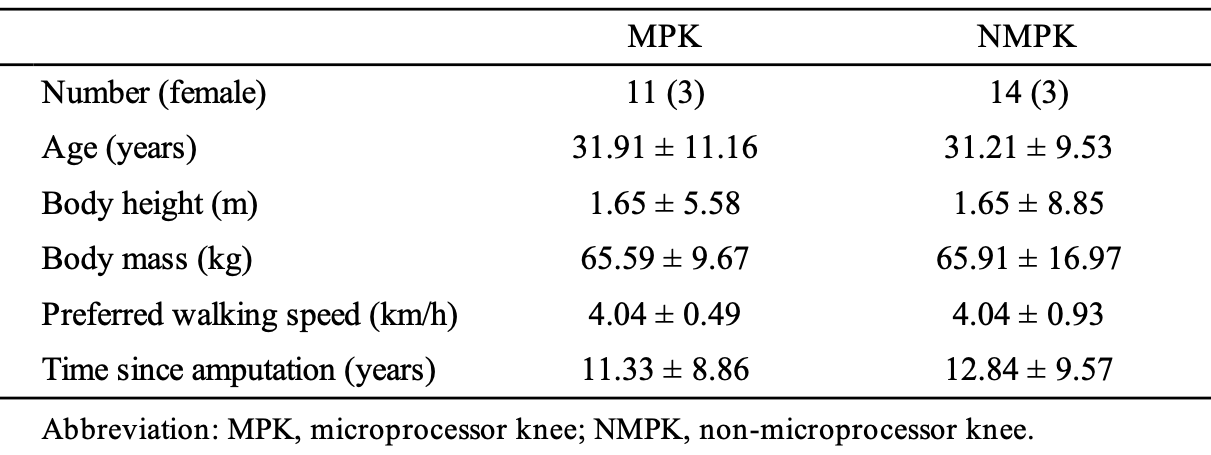


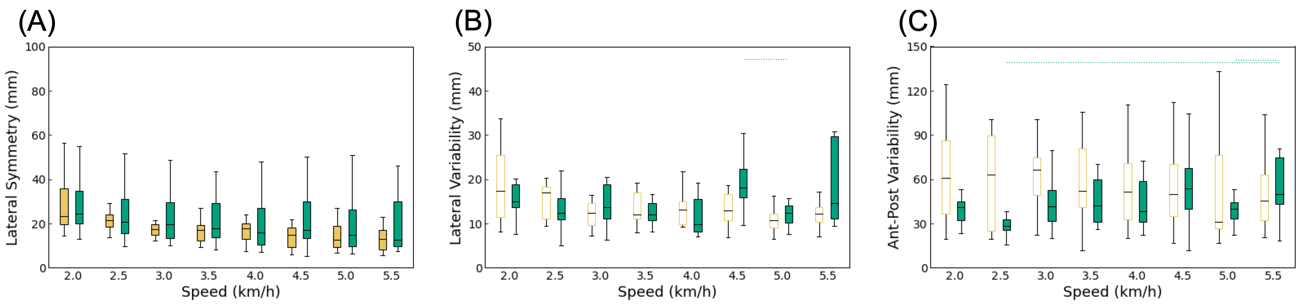


**Figure S1**. Whisker-box plots of lateral symmetry (A), lateral variability (B), and anterior–posterior variability (C) at eight different walking speeds. The yellow and green boxes correspond to MPK and NMPK users, respectively. There are no significant differences in Lateral symmetry, Lateral variability, and Anterior-posterior variability between the two groups over a wide range of walking speeds. Significant and non-significant main effects of walking speeds are indicated by filled and unfilled boxes, respectively. The green (NMPK users) horizontal lines indicate significant differences between walking speeds (dotted line: p < 0.05, solid line: p < 0.01).
